# Innovations and Challenges in Smart-Textile Wearables for Postural Monitoring: A Scoping Review

**DOI:** 10.3390/jcm15218478

**Published:** 2026-10-30

**Authors:** Inês Peixoto, Carla Silvia Fernandes, Marta Campos Ferreira

**Affiliations:** 1 Department of Industrial Engineering and Management, Faculdade de Engenharia da Universidade do Porto, 4200-465 Porto, Portugal; up202106794@edu.fe.up.pt; 2 School of Health, Polytechnic Institute of Viana do Castelo, 4910-023 Viana do Castelo, Portugal; fernandes.carla@ess.ipvc.pt; 3 Health Sciences Research Unit: Nursing (UICISA:E), 3046-851 Coimbra, Portugal; 4 ADITGames Association, 4490-010 Póvoa de Varzim, Portugal; 5 INESC TEC, Faculdade de Engenharia da Universidade do Porto, 4200-46 Porto, Portugal

**Keywords:** wearable technology, smart textiles, posture monitoring, inertial measurement unit (IMU), energy harvesting, work-related musculoskeletal disorders (WRMSD)

## Abstract

In many work environments, professionals perform physically demanding routines involving repetitive movements and prolonged static postures, increasing exposure to biomechanical risk factors associated with work-related musculoskeletal disorders (WRMSDs). Wearable technologies offer opportunities for continuous posture monitoring and real-time feedback, but their occupational deployment remains challenging due to limitations related to comfort, durability, energy autonomy, and integration with work clothing. This scoping review aims to synthesize and critically analyze the existing evidence on smart-textile-based wearable systems for occupational posture monitoring. Following the PRISMA-ScR framework, a systematic search of Scopus, Web of Science, PubMed, and CINAHL was conducted, identifying 566 records. After screening and eligibility assessment, 34 studies were included in the final synthesis. The included studies were analyzed according to wearable architectures, sensing technologies, data processing and posture recognition, user feedback mechanisms, and energy management strategies. Hybrid architectures combining textile-based sensors and inertial measurement units, together with machine learning and real-time feedback, emerged as promising approaches for continuous posture monitoring. However, the evidence was predominantly based on technical or laboratory validation, with relatively small samples and limited long-term evaluation in real occupational environments. Five studies reported changes in posture or ergonomic behavior following the use of the technology, while no direct evidence of reductions in WRMSD incidence or severity was identified. Overall, the findings demonstrate substantial technological potential for smart-textile-based posture monitoring, but current evidence is insufficient to establish clinical or occupational effectiveness in preventing WRMSDs. Future research should prioritize prospective, long-term validation involving larger and more diverse worker populations under real-world occupational conditions, while addressing comfort, durability, energy autonomy, and compatibility with occupational clothing and personal protective equipment.

## 1. Introduction

Work-related musculoskeletal disorders (WRMSDs) are among the most prevalent occupational health problems worldwide, affecting an estimated 1.71 billion people globally and nearly three out of five workers in the European Union [1,2]. They represent a major challenge for both workers and organizations, contributing to pain, functional limitations, reduced quality of life, absenteeism, and substantial healthcare and productivity costs [3]. The development of WRMSDs is multifactorial, resulting from the interaction of biomechanical, organizational, psychosocial, and individual factors. Among the most significant occupational risk factors are repetitive movements, prolonged awkward postures, manual material handling, and exposure to mechanical vibrations, all of which increase mechanical stress on the musculoskeletal system [2,4].

The prevention and assessment of WRMSDs have traditionally relied on observational ergonomic methods and self-reported questionnaires [5]. Although these approaches remain widely used in occupational health practice, they are inherently subjective, depend on evaluator expertise, and provide only periodic assessments rather than continuous monitoring [6]. Laboratory-based motion capture systems offer considerably higher accuracy for biomechanical analysis but require sophisticated infrastructure, extensive calibration, and controlled experimental environments, limiting their applicability in real workplaces characterized by dynamic tasks, restricted spaces, and variable environmental conditions [7]. These limitations have motivated the search for monitoring technologies capable of providing objective, continuous, and unobtrusive ergonomic assessment during daily work activities.

Recent advances in wearable sensing technologies have created new opportunities for continuous posture monitoring and early prevention of WRMSDs. In particular, smart textiles integrate sensing elements directly into garments, enabling the acquisition of biomechanical data while preserving worker comfort and freedom of movement [8]. By embedding inertial, strain, pressure, or textile-based sensors into fabrics, these systems allow posture and movement to be monitored throughout normal work activities with minimal interference [9]. Beyond passive monitoring, wearable systems can also provide real-time feedback when hazardous postures are detected. Among the different feedback modalities, vibrotactile feedback has attracted particular attention because it is discreet, does not require visual attention, and is less affected by noisy occupational environments than auditory alerts, making it well suited for industrial and healthcare settings [2,8].

In particular, smart textiles have emerged as a promising approach for unobtrusive wearable monitoring. *Smart textiles* are textile-based materials or garments incorporating sensing, processing, communication, or other electronic functionalities, enabling physiological or biomechanical information to be acquired while preserving the flexibility and wearability of conventional textiles. In the context of this review, *smart-textile wearables* refer specifically to body-worn systems in which sensing or related electronic components are integrated into, attached to, or embedded within textile-based structures. By embedding inertial, strain, pressure, or textile-based sensors into fabrics, these systems allow posture and movement to be monitored throughout normal work activities with minimal interference with the user’s movements and comfort [9].

Despite these advantages, several technical challenges continue to limit the widespread adoption of smart wearable systems. One of the most significant is energy autonomy [10]. Most existing devices rely on conventional batteries, whose limited lifetime requires frequent charging or replacement, reducing usability and increasing maintenance requirements [11]. In some occupational environments, such as mining, construction, or heavy industry, batteries may also pose safety concerns associated with overheating or fire hazards [12]. Consequently, considerable research has focused on energy-efficient system design and energy harvesting technologies capable of converting ambient or human-generated energy into electrical power. Among these approaches, piezoelectric energy harvesting has emerged as a promising solution for wearable applications by converting mechanical energy generated during human movement or whole-body vibration into usable electrical energy [11]. Flexible piezoelectric polymers, such as polyvinylidene fluoride (PVDF), are particularly attractive because they combine low weight, flexibility, durability, and user comfort, making them suitable for integration into smart garments and personal protective equipment [12].

Several review studies have synthesized the broader literature on WRMSDs, wearable sensing, and posture or motion monitoring. These reviews have contributed important insights into occupational risk assessment, wearable sensor technologies, inertial sensing, textile-based sensing, and machine learning approaches for human activity and posture recognition [13,14,15,16,17,18,19]. However, their scopes differ considerably. Reviews addressing WRMSDs have predominantly focused on risk factors, assessment methods, prevention strategies, or specific occupational populations, rather than on the technological characteristics of wearable systems for continuous postural monitoring. Conversely, reviews of wearable technologies have generally considered heterogeneous wearable devices, without systematically distinguishing smart-textile-based systems from other wearable form factors. Similarly, reviews focused on specific sensing modalities have often examined sensor performance or recognition algorithms without integrating garment design, user feedback, and energy autonomy into a single framework.

As a result, important gaps remain in the current literature, which this scoping review specifically addresses. First, there is a lack of a comprehensive synthesis specifically addressing smart-textile-based wearable systems designed for occupational posture monitoring and their potential role in supporting WRMSD prevention. Second, the evidence is fragmented across sensing technologies, textile integration strategies, sensor placement, data processing and posture recognition methods, and feedback mechanisms, making it difficult to understand how these technological components are combined within complete wearable systems. Third, energy management and energy harvesting, despite being critical to the autonomy and practical deployment of wearable devices, have received limited attention as an integrated component of smart-textile postural monitoring systems. Finally, many reported systems have been evaluated in controlled laboratory settings with relatively small or homogeneous samples, leaving uncertainty regarding their robustness, usability, comfort, and applicability in real occupational environments. By bringing these dimensions together, the present review extends previous reviews by focusing specifically on smart-textile-based wearable systems and by examining their technological components as an integrated framework for occupational posture monitoring.

From an occupational and clinical medicine perspective, addressing these gaps is important because reliable postural monitoring may support the identification of excessive or prolonged biomechanical exposure before symptoms or functional limitations become clinically apparent. Such information may complement conventional ergonomic assessments and facilitate more timely preventive interventions, while also providing clinicians, occupational health professionals, and employers with objective information to support workplace risk management. The integration of continuous monitoring, real-time feedback, and wearable textile technologies may therefore contribute to a more proactive approach to occupational health, while recognizing that technological monitoring alone cannot replace comprehensive ergonomic assessment or organizational prevention measures.

These gaps motivate the need for a focused scoping review that goes beyond identifying wearable sensing technologies and instead examines smart-textile wearables as integrated systems for occupational postural monitoring. In particular, understanding the relationship between sensing technology, garment integration, computational processing, feedback, and energy management is essential for identifying the technological barriers that currently limit the transition from experimental prototypes to practical workplace applications.

Accordingly, this scoping review addresses the following research questions (RQs):

- **RQ1:** What sensing technologies and smart-textile integration strategies have been used for occupational posture monitoring?
- **RQ2:** What data processing, posture recognition, and classification methods have been employed in smart-textile-based wearable systems?
- **RQ3:** What feedback mechanisms have been incorporated to support real-time detection and prevention of potentially hazardous postures?
- **RQ4:** What energy management and energy harvesting approaches have been investigated to improve the autonomy of smart-textile wearable systems?
- **RQ5:** What are the main technological, usability, and validation challenges limiting the application of these systems in real-world occupational environments?

To address these questions, this scoping review aims to identify, classify, and critically analyze smart-textile-based wearable systems designed for posture monitoring in occupational contexts. Specifically, the review examines the sensing technologies employed, garment integration strategies, data processing and posture recognition methods, user feedback mechanisms, and energy management solutions, including energy harvesting approaches. By synthesizing these dimensions within a unified framework, this review seeks to clarify the current state of the art, identify the main technological and methodological gaps, and highlight opportunities for future development toward comfortable, autonomous, and effective wearable systems capable of supporting the primary prevention of WRMSDs in real-world working environments.

## 2. Methodology

The methodology was designed to ensure a transparent, systematic, and reproducible review process. The following subsections describe the review design, literature search and eligibility criteria adopted to identify and analyze the relevant evidence. Then, the study selection process, data extraction, and synthesis are presented.

### 2.1. Review Design

This study was conducted as a scoping review addressing smart-textile wearable technologies developed for postural monitoring in environments exposed to biomechanical risk factors associated with work-related musculoskeletal disorders (WRMSDs). The review was conducted following the methodological guidance for scoping reviews developed by the Joanna Briggs Institute (JBI) [20]. The Population, Concept, and Context (PCC) framework was adopted to structure the review question and search strategy [20]. The reporting of the review followed the Preferred Reporting Items for Systematic Reviews and Meta-Analyses extension for Scoping Reviews (PRISMA-ScR) [21].

Accordingly, the Population was defined as workers or individuals engaged in occupational or work-like physical activities. The Concept focused on smart textiles and wearable systems based on textile-integrated sensors for posture and movement monitoring. The Context included work-related musculoskeletal disorders, postural risk, ergonomic hazards, and occupational environments in which prolonged, repetitive, or inadequate postures may contribute to injury [1,2].

### 2.2. Data Sources

To ensure broad coverage of relevant studies across health, engineering, and technology domains, searches were conducted in four major databases: Scopus, Web of Science, PubMed, and Cumulative Index to Nursing and Allied Health Literature (CINAHL). These sources were selected to capture both multidisciplinary and specialized literature. Scopus and Web of Science provided wide coverage of engineering and technology research, while PubMed and CINAHL contributed a stronger focus on health, ergonomics, and occupational safety.

### 2.3. Search Strategy

The primary literature search across the selected databases was conducted in October 2025. The search strategy was developed iteratively using the PCC approach, combining controlled vocabulary with free-text keywords. To ensure a comprehensive retrieval of relevant studies, the search string was specifically applied to the Title, Abstract, and Keywords fields of the indexed documents. No automated restrictions (such as publication year or language limits) were initially applied at the database level; instead, the search relied entirely on the strictness of the Boolean operators within the query to filter the results, allowing for a broad initial capture of the literature before the manual screening phase.

The search strategy was adapted to the syntax and indexing characteristics of each database. The complete search strategies used for Scopus, Web of Science, PubMed, and CINAHL are provided in Appendix A.

The database search retrieved a total of 566 records, including 334 from Scopus, 200 from Web of Science, 18 from PubMed, and 14 from CINAHL. After merging the records, duplicate removal was performed using the Rayyan platform, resulting in 371 unique studies for screening.

### 2.4. Eligibility Criteria

Eligibility criteria were defined *a priori* to ensure consistency with the objectives of the review. Studies were eligible if they: (i) investigated smart textiles or related technologies, such as e-textiles, smart garments, or textile-based sensors; (ii) focused on posture monitoring, postural assessment, or movement-related monitoring relevant to ergonomic risk; (iii) described wearable systems intended to be worn directly on the body rather than integrated into external objects; and (iv) reported an experimental, validation, prototype-development, or application-oriented study. Peer-reviewed journal articles and full conference papers were considered eligible, whereas review articles, editorials, commentaries, conference abstracts, and other non-primary publications were excluded.

Regarding the occupational context, studies were considered eligible when their systems addressed occupational activities, work-related postural or movement patterns, or biomechanical conditions that are relevant to occupational ergonomic assessment. For studies conducted in non-occupational contexts, eligibility was determined based on the specific postures, movements, or biomechanical patterns investigated and their applicability to occupational ergonomic assessment, rather than on the activity itself. This included studies where the monitored movements involved body postures, trunk inclination, upper-limb movements, or other biomechanical patterns that are also relevant to occupational tasks. The rationale for including studies conducted in non-occupational contexts is reported in Appendix B. Studies focused exclusively on non-occupational activities or contexts, without a clear relevance to occupational posture monitoring or ergonomic risk, were excluded.

Only studies published in English from 2015 onwards were considered eligible. The restriction to English was adopted to ensure consistent interpretation and critical assessment of the included studies within the scope and resources of the review. The publication period was selected to capture the recent development and technological evolution of smart textiles, wearable sensing technologies, and posture-monitoring systems, while excluding earlier literature that may reflect substantially different technological architectures and levels of integration. In addition to the electronic database search, the reference lists of key articles and relevant review papers were manually screened to identify potentially eligible studies not retrieved through the database search. It should be noted that review articles identified during the search and reference-list screening were used only as background sources and were not included among the 34 studies in the final synthesis. Also, peer-reviewed journal articles and full papers published in conference proceedings were considered eligible. Conference abstracts, extended abstracts without a full paper, review articles, editorials, commentaries, and other non-primary publications were excluded. Table 1 summarizes the inclusion and exclusion criteria adopted in this review.

### 2.5. Study Selection

After duplicate removal, the remaining 371 records were screened. During this initial stage, both titles and abstracts were concurrently analyzed to assess their relevance to the research objectives. The titles and abstracts were independently screened by the three reviewers according to the predefined eligibility criteria. Disagreements between reviewers were discussed and resolved through consensus. This combined screening process resulted in the exclusion of 298 articles, leaving 73 articles for full-text evaluation.

**Figure 1:**
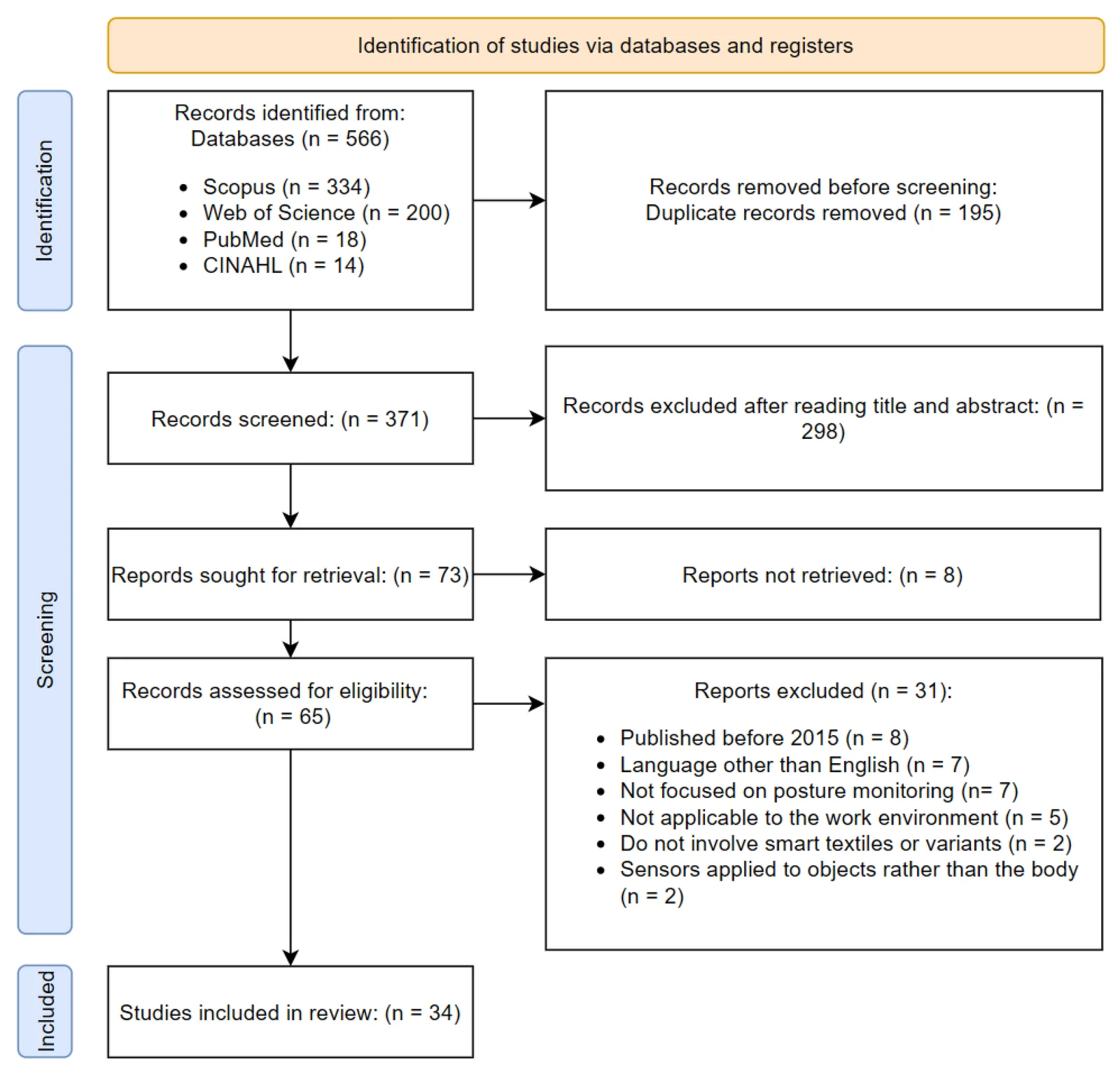
PRISMA-ScR flow diagram of the study selection process.

The full texts of 73 articles were sought for eligibility assessment. Despite repeated attempts to obtain the complete manuscripts through institutional library resources, publisher websites, and other available channels, the full texts of eight articles could not be accessed and were therefore excluded from further assessment. The remaining 65 articles underwent full-text eligibility screening, after which 34 studies were included in the final synthesis and 31 were excluded for not meeting the review objectives or eligibility criteria. Full-text eligibility was independently assessed by the three reviewers, with disagreements discussed and resolved through consensus. The complete study selection process is illustrated in the PRISMA-ScR flow diagram shown in Figure 1.

### 2.6. Data Extraction and Synthesis

For each included study, data were extracted by one reviewer using a structured template designed to capture the main technical and methodological characteristics of the proposed systems. The extracted information included study identification, wearable architecture, sensor technologies, placement and integration strategy, data acquisition procedures, posture recognition methods, user feedback mechanisms, and energy management approaches. The extracted data were subsequently independently checked by the other two reviewers. Any discrepancies identified during the checking process were discussed among the three reviewers and resolved through consensus.

Because the included studies were highly heterogeneous in terms of device design, sensing modalities, experimental protocols, and outcome measures, a quantitative meta-analysis was not considered appropriate. Instead, the evidence was synthesized descriptively and thematically, allowing the literature to be organized into key technical domains relevant to both wearable system design and WRMSD prevention.

Given the scoping review design and the objective of mapping and characterizing the available evidence, a formal methodological quality or risk-of-bias assessment was not performed. The purpose of this review was to map the range and characteristics of smart-textile-based wearable systems for occupational posture monitoring rather than to estimate intervention effectiveness or determine the certainty of evidence. Therefore, methodological quality was not used as an eligibility or exclusion criterion. Nevertheless, relevant methodological characteristics of the included studies were extracted and considered when interpreting and synthesizing the evidence.

## 3. Results

The results are presented as a descriptive analysis of the 34 included studies, followed by a thematic synthesis of wearable architectures, sensor technologies, data processing and posture recognition, user feedback, and energy management. Study-level characteristics are provided in Appendix B.

### 3.1. Descriptive Analysis

**Figure 2:**
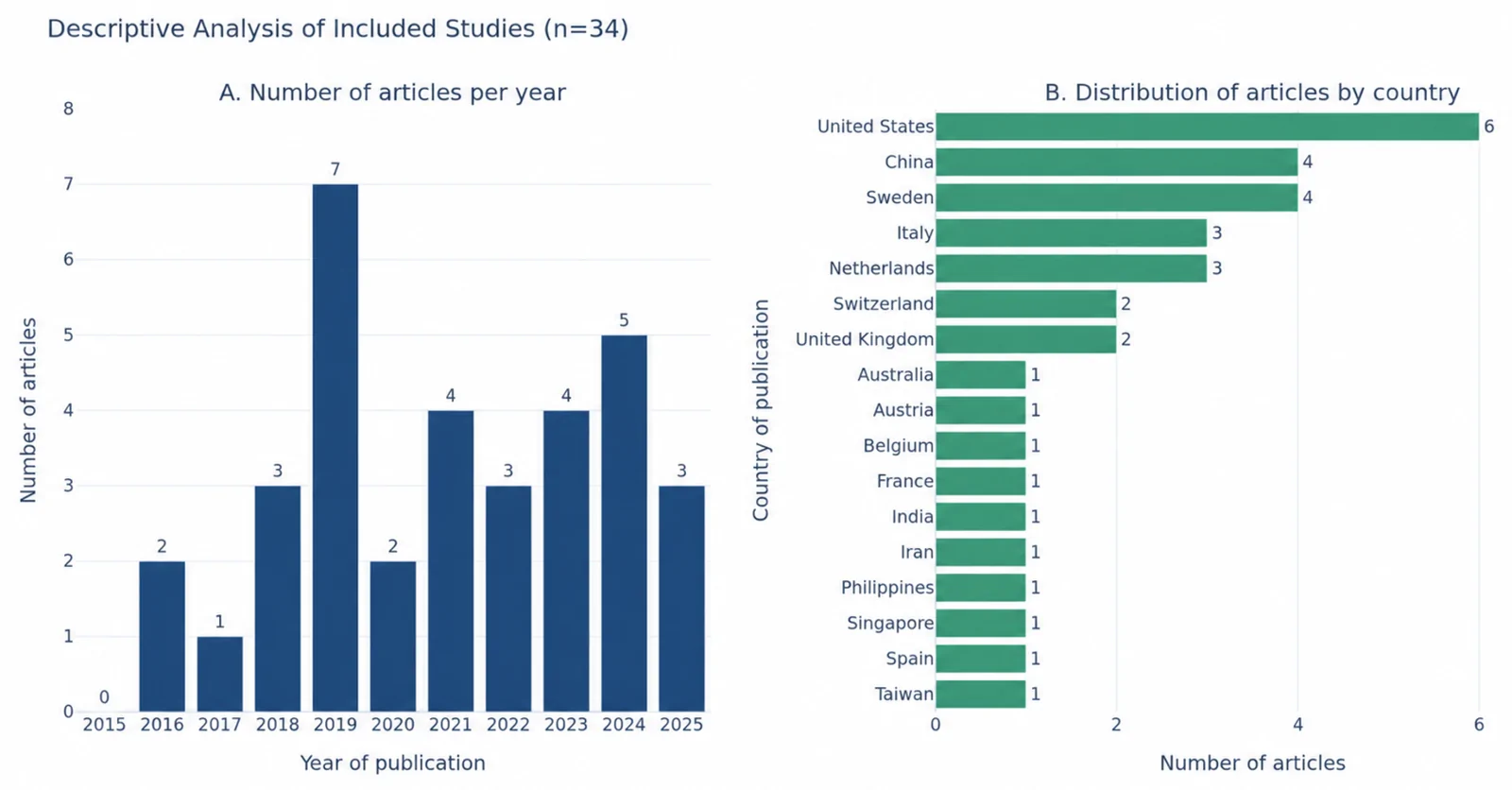
Descriptive synthesis of the included studies (n=34): (**A**) Number of articles published per year; (**B**) Distribution of articles by country of publication.

Figure 2 presents the distribution of the 34 included studies by publication year and country. The highest annual number of publications was recorded in 2019 (n=7). The United States accounted for six studies (17.65%), followed by China and Sweden with four each (11.76% each), Italy and the Netherlands with three each (8.82% each), and Switzerland and the United Kingdom with two each. The remaining countries contributed one study each.

### 3.2. Thematic Analysis of the Included Studies

**Figure 3:**
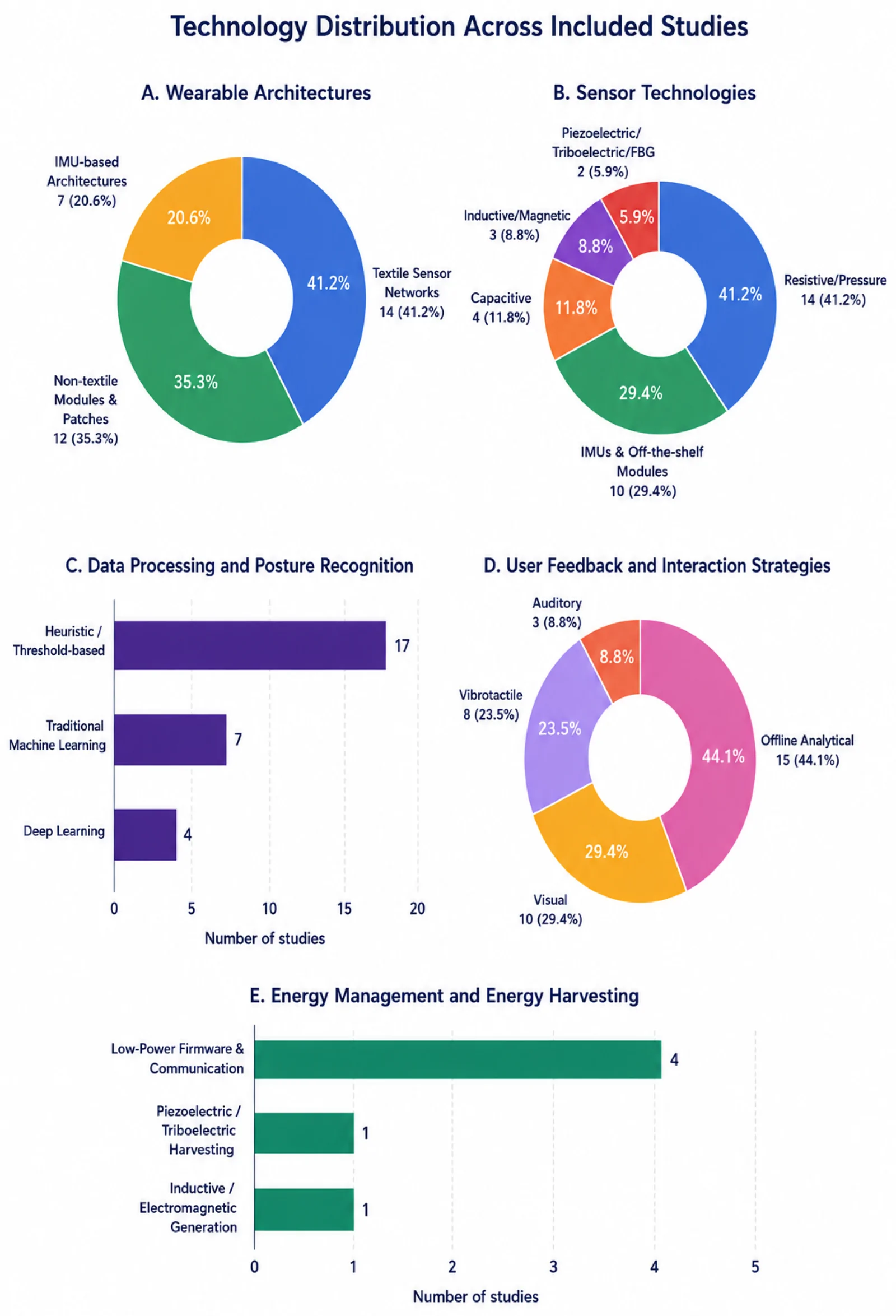
Technical synthesis of the 34 included studies. The panels display the distribution of the adopted approaches categorized by: (**A**) wearable architectures adopted for postural monitoring; (**B**) sensor technologies; (**C**) data processing and posture recognition methodologies; (**D**) user feedback modalities; and (**E**) energy management strategies. Note that panels (**C**,**E**) include studies employing multiple approaches simultaneously.

The included studies were organized into five themes: wearable architectures, sensor technologies, data processing and posture recognition, user feedback and interaction strategies, and energy management and energy harvesting. Figure 3 summarizes the classifications across these themes. Individual study characteristics are reported in Appendix B.

Five studies reported an evaluation of changes in posture or ergonomic behavior following use of the wearable system [16,22,23,24,25]. No included study directly evaluated WRMSD-related clinical outcomes.

The counting rules differ by theme: the wearable architecture and sensor technology tables assign each study to one category, whereas studies may be represented in multiple categories in the data processing, feedback, and energy management analyses where multiple approaches were reported. Studies not reporting a given characteristic are not assigned to a category for that theme. The number of distinct studies and any unclassified studies should therefore be considered separately from the sum of category counts.

### 3.3. Wearable Architectures for Postural Monitoring

The wearable architectures were classified into three mutually exclusive groups (Table 2): textile sensor networks (n=15), non-textile modules and patch integrations (n=12), and inertial modules and rigid electronics (n=7). Each of the 34 included studies was assigned to one architectural family. The systems included textile-integrated sensing elements, garment-mounted inertial modules, and wearable patches or other modular sensor assemblies.

### 3.4. Sensor Technologies

Five sensor technology families were identified (Table 3). Resistive and pressure sensors were classified in 14 studies, IMUs and off-the-shelf modules in 11, capacitive sensors in four, inductive and magnetic sensors in three, and piezoelectric, triboelectric, or fiber Bragg grating sensors in two. Each study was assigned to one category in this table. Reported implementations included conductive textile structures, pressure-sensing matrices, capacitive patches, coils or resonant elements, optical sensing elements, and inertial modules.

### 3.5. Data Processing Algorithms and Posture Recognition

The reported data processing approaches were grouped into heuristic and threshold-based methods (n=17), traditional machine learning (n=7), and deep learning (n=4), as shown in Table 4. Heuristic approaches included thresholds, kinematic calculations, and rule-based procedures. Traditional machine learning methods included support vector machines, k-nearest neighbors, random forests, and artificial neural networks; deep learning implementations included convolutional and recurrent neural network architectures. The table lists the studies classified under each approach; six of the 34 studies are not represented in these three categories.

### 3.6. User Feedback and Interaction Strategies

Four feedback modalities were identified (Table 5): offline analytical feedback (n=15), visual feedback (n=10), vibrotactile feedback (n=8), and auditory feedback (n=3). Visual interfaces included mobile applications and dashboards; vibrotactile and auditory approaches used physical or sound notifications, while offline analytical approaches involved subsequent review of recorded measurements. Some studies reported more than one feedback modality and are therefore listed in multiple rows.

### 3.7. Energy Management and Energy Harvesting

Explicit energy management or harvesting strategies were identified in six studies (Table 6). Four studies reported low-power firmware or communication approaches, including sampling-rate adjustments, local processing, and communication management [17,32,45,47]. One study was classified under piezoelectric or triboelectric harvesting [12], and one under inductive or electromagnetic generation [13]. The other 28 included studies were not assigned to these energy-strategy categories.

## 4. Discussion and Identified Gaps

This scoping review mapped the architectures, sensing technologies, posture-recognition approaches, user feedback mechanisms, and energy-management strategies reported in 34 studies of smart textile-based wearable systems. The findings show a diverse set of technological solutions, but the evidence is predominantly concerned with prototype development and technical validation. The following discussion interprets these patterns in relation to occupational ergonomic monitoring, distinguishes demonstrated performance from potential applications, and identifies priorities for further research.

### 4.1. Synthesis of Current Evidence

A central finding is the distinction between successful posture detection and demonstrated occupational health benefits. Many included studies evaluated sensing performance or posture-classification accuracy under specified experimental conditions, whereas relatively few examined changes in users’ posture or ergonomic behaviour following system use [16,22,23,24,25]. The reviewed evidence does not establish that these systems reduce the incidence or severity of work-related musculoskeletal disorders (WRMSDs). High classification accuracy should therefore be understood as a measure of technical performance, not as proof of preventive effectiveness.

The architectural categories reflect different design priorities rather than a hierarchy of effectiveness. Systems incorporating inertial measurement units (IMUs) can measure orientation and movement across body segments using established sensing components [17,26,27,28]. Their integration into garments, however, introduces practical considerations relating to attachment, bulk, power supply, and user comfort. Textile-integrated sensors can distribute measurements across flexible garment surfaces and reduce the need for separate attachments [14,18,29,34]. At the same time, textile sensing may require careful calibration and attention to deformation-related variability, signal drift, and durability [14,34,38]. Modular and patch-based approaches broaden the available sensing options, but their suitability for sustained occupational use depends on how the components are positioned and secured. These trade-offs suggest that the preferred architecture should be selected according to the body regions monitored, target tasks, measurement requirements, and conditions of use, rather than assuming that one family is universally preferable.

The sensor technologies identified in the review also differ in what they measure. Resistive and pressure-based elements capture local contact or deformation, whereas inertial sensors provide information on segment orientation and motion. Capacitive, inductive, and other sensing principles offer additional ways of measuring deformation or movement [13,33,39,40]. Combining complementary sensing modalities may help address limitations of individual measurements, but the reviewed studies do not provide a sufficiently consistent basis to conclude that hybrid systems are generally more accurate, comfortable, or energy-efficient. Comparative evaluations under equivalent occupational conditions are needed to establish such benefits.

Posture-recognition methods ranged from threshold-based and heuristic approaches to conventional machine learning and deep learning. This diversity reflects differences in sensing configurations, target postures, data availability, and processing requirements. Conventional classifiers can perform well on defined posture-recognition tasks [16,17,18,47], while deep learning approaches have been investigated for more complex sensor patterns and temporal information [14,27,34,36]. However, reported accuracy values cannot be compared directly when studies use different participant samples, activity sets, validation protocols, and outcome measures. For occupational deployment, computational demands, inter-user generalizability, calibration needs, and the feasibility of on-device processing should be considered alongside classification performance.

Feedback is another dimension in which technical feasibility must be distinguished from demonstrated behavioural impact. Visual, auditory, and vibrotactile mechanisms provide different ways of communicating posture-related information to users [16,22,24]. Vibrotactile cues may be useful when visual attention is occupied, whereas visual interfaces can support retrospective inspection of monitored data. Nevertheless, the included evidence does not support a general conclusion that one feedback modality is consistently more effective than another, or that real-time feedback necessarily produces sustained ergonomic improvements. Such claims require comparative studies that assess behaviour over time and account for the characteristics of specific workplaces.

Energy management was less consistently reported than sensing and posture recognition. This limits the extent to which the practical autonomy of different systems can be compared. Energy harvesting and low-power design approaches have been investigated in the literature [12], but their contribution to complete-system operation during a working shift remains insufficiently documented in the included studies. Future evaluations should report battery capacity, power consumption, sampling and communication settings, processing workload, and achieved operating time using comparable measurement procedures.

Finally, the included studies varied in their proximity to occupational practice. Some evaluated work-related tasks directly, whereas others investigated postures or movements in non-occupational contexts. The latter studies contribute information on sensing capabilities and technical transferability, but should not be interpreted as direct evidence of performance or effectiveness in workplaces. This distinction is particularly important when translating results from controlled activities to complex, prolonged, and variable occupational tasks.

### 4.2. Research Gaps and Future Research Opportunities

The first research gap concerns the integration of system components. The literature includes numerous examples of individual sensing elements, recognition methods, and feedback mechanisms, but fewer evaluations of complete systems combining these functions with documented energy autonomy and long-term wearability. Future work should assess the performance of the full wearable system rather than treating sensor accuracy, user interaction, and power consumption as independent design problems.

A second gap concerns validation conditions and external validity. Many studies relied on laboratory experiments, predefined postures, or limited participant samples [18,30,40,42,47]. Although these designs are appropriate for early technical development, they do not necessarily represent the variability of real work. Field evaluations, including studies in occupational settings such as that reported by [45], should be extended to different tasks, body types, clothing configurations, and environmental conditions. Independent validation and transparent reporting of participant-level data splits would also improve the interpretability of posture-recognition performance.

A third gap concerns long-term usability and textile durability. Comfort and acceptance measured during short tests may not predict sustained use across full working shifts. Repeated movement, perspiration, laundering, and wear can also influence sensing materials and electrical connections [14,34,37,38]. Future studies should therefore assess calibration stability, washability, maintenance requirements, and user acceptance over extended periods, including compatibility with occupational clothing and personal protective equipment.

A fourth gap is the limited evidence linking monitoring and feedback to occupational outcomes. Research should distinguish between accurate recognition of a posture, a short-term behavioural response to feedback, sustained reductions in biomechanical exposure, and longer-term health outcomes. Prospective evaluations with appropriate comparison conditions are needed to determine whether wearable monitoring contributes to meaningful ergonomic improvements and, ultimately, whether it affects WRMSD-related outcomes.

Finally, energy autonomy needs more systematic evaluation. Reporting should extend beyond individual sensor characteristics to the power requirements of the complete sensing, processing, communication, and feedback chain. Comparisons between local processing and wireless transmission, as well as assessments of energy-harvesting contributions, should be based on measured operating conditions rather than assumed benefits.

### 4.3. Towards Next-Generation Smart Textile Wearable Systems

The identified gaps suggest that future systems would benefit from a more integrated, user-centred design process. Sensor selection should be guided by the biomechanical variables and occupational tasks of interest. Hybrid combinations of textile and inertial sensing are a plausible option where local deformation and segment motion provide complementary information, but their added value should be demonstrated through direct comparison with simpler configurations [13,17,28].

Similarly, algorithm selection should consider recognition performance together with processing requirements, user-specific calibration, and robustness to changing conditions. On-device inference may reduce reliance on continuous data transmission in some designs, but its effect on total energy consumption and responsiveness should be measured. Feedback mechanisms should be selected and evaluated with workers, considering task demands, acceptability, potential distraction, and whether any behavioural change persists after initial use [16,22,25].

Energy autonomy, durability, and integration with workwear should be addressed from the earliest design stages. Rather than assuming that energy harvesting or any particular wireless protocol will provide sufficient autonomy, developers should quantify complete-system power requirements under realistic usage patterns. Taken together, these considerations point towards systems evaluated not only for technical feasibility but also for sustained usability and measurable relevance to occupational ergonomic assessment.

### 4.4. Practical Implications

For researchers and developers, the findings emphasize the importance of reporting comparable technical and operational metrics, including sensing accuracy, validation design, calibration procedures, operating time, comfort, and durability. For occupational health professionals, wearable monitoring may complement observational assessments by providing additional information on postures and movements during selected activities. However, the selection of a system should be based on the specific assessment objective, workplace conditions, and the strength of its validation evidence.

Organizations considering deployment should also account for compatibility with workwear and personal protective equipment, maintenance and laundering requirements, worker acceptance, and the interpretation of generated data. The current evidence does not justify treating posture-recognition performance as proof of WRMSD prevention. Wearable monitoring should therefore be considered a potential support for ergonomic assessment and intervention, rather than a substitute for established occupational risk-management practices.

### 4.5. Limitations of This Review

This review has several limitations. The included studies were heterogeneous in their architectures, sensing principles, target body regions, participants, experimental tasks, and performance measures. As a result, direct comparisons between reported performance values were limited, and a quantitative synthesis of effectiveness was not undertaken.

The review was restricted to English-language publications from 2015 onwards. Relevant work published in other languages or before this period may therefore have been missed. In addition, eight records could not be included because their full texts were unavailable despite retrieval attempts, introducing a possible source of selection bias.

As a scoping review, the study aimed to map technological characteristics and research gaps rather than estimate intervention effects. A formal risk-of-bias assessment was not used to determine study inclusion, and the synthesis should not be interpreted as evidence of comparative or clinical effectiveness. Moreover, some included studies were conducted outside occupational settings because their monitored biomechanical variables or sensing approaches were potentially transferable to occupational assessment. Such transferability remains a hypothesis unless validated in relevant workplace conditions.

Finally, the conclusions are constrained by the predominance of prototype and short-duration validation studies in the available literature. Evidence concerning sustained changes in ergonomic behaviour and WRMSD-related health outcomes remains limited. These uncertainties should be considered when interpreting the potential practical value of the reviewed technologies.

## 5. Conclusions

This scoping review provides a comprehensive overview of the current state of the art in smart textile-based wearable systems for posture monitoring aimed at preventing work-related musculoskeletal disorders (WRMSDs). Following the PRISMA-ScR framework, 34 studies were systematically analyzed to examine wearable architectures, sensing technologies, data processing approaches, user feedback mechanisms, and energy management strategies.

The findings indicate that recent research has made significant progress toward the development of wearable systems capable of continuously monitoring posture in occupational environments. Overall, the reviewed evidence suggests that hybrid architectures integrating textile-based sensors with inertial measurement units (IMUs), lightweight edge-computing algorithms, and real-time vibrotactile feedback represent promising solutions for combining sensing capabilities, user comfort, and computational efficiency. Nevertheless, energy management remains one of the least explored aspects of current systems, highlighting the need for greater integration of low-power design principles and energy harvesting technologies to support prolonged autonomous operation.

Despite these advances, several important research gaps remain. Most studies are based on laboratory experiments involving relatively small and homogeneous participant samples, limiting the generalizability of their findings to real occupational settings. Furthermore, long-term field validation, comprehensive assessments of usability and user acceptance, and systematic characterization of energy autonomy are still largely lacking. Importantly, although a small number of studies reported improvements in posture or ergonomic behavior, the reviewed literature did not provide direct evidence that smart-textile wearable systems reduce the incidence or severity of WRMSDs. The current evidence therefore demonstrates substantial technological potential, but their occupational and clinical effectiveness remains to be established.

By synthesizing the current evidence and identifying the principal technological and methodological gaps, this review provides a foundation for the design of next-generation smart textile wearables. Future research should prioritize integrated, user-centred systems that combine comfort, reliability, energy efficiency, and robust validation in real occupational environments. In particular, prospective and long-term studies involving larger and more diverse worker populations are needed to assess performance under realistic working conditions, evaluate sustained changes in ergonomic behavior, and determine whether these technologies can ultimately contribute to measurable improvements in occupational health outcomes and reductions in WRMSD incidence or severity.

## Figures and Tables

**Table 1:** Inclusion and exclusion criteria.

Inclusion Criteria	Exclusion Criteria
Investigate smart textiles or related variants, such as e-textiles, smart garments, or textile-based sensors. Focused on posture, postural assessment, or movement monitoring relevant to ergonomic risk. Wearable systems intended to be worn directly on the body. Address occupational activities, work-related postures, or movements relevant to occupational ergonomic assessment. Experimental, validation, prototype-development, or application-oriented primary studies. Peer-reviewed journal articles or full conference papers. Written in English. Published from 2015 onwards.	Not focused on posture or movement monitoring. Not relevant to occupational posture monitoring or ergonomic risk. Sensors applied to objects rather than the body. Do not involve smart textiles or related variants. Review articles, editorials, commentaries, conference abstracts, or other non-primary publications. No access to full text. Not in English. Published before 2015.

**Table 2:** Summary of Wearable Architectures identified in the literature, categorized by technological family.

Architectural Family	References from Included Studies	Total Studies
**Inertial Modules (IMUs) and Rigid Electronics**	[17,22,24,25,26,27,28]	7
**Textile Sensor Networks (e.g., Piezoresistive, Mesh)**	[14,15,16,18,29,30,31,32,33,34,35,36,37,38,39]	15
**Non-textile Modules & Patch Integrations**	[12,13,23,40,41,42,43,44,45,46,47,48]	12

**Table 3:** Summary of Sensor Technologies identified in the literature, categorized by operating principle.

Sensor Technology Family	References from Included Studies	Total Studies
**Resistive and Pressure Sensors** (e.g., Piezoresistive, Conductive Threads)	[14,15,16,18,29,30,31,32,34,36,37,38,46,47]	14
**Capacitive Sensors** (e.g., Dielectric Stacks, Capacitive Patches)	[23,33,43,44]	4
**Inductive and Magnetic Sensors** (e.g., Coils, Resonant Structures)	[13,39,40]	3
**Piezoelectric, Triboelectric & FBG** (e.g., Vibration, Advanced Optical Fibers)	[12,42]	2
**IMUs and Off-the-shelf Modules** (e.g., Accelerometers, Gyroscopes)	[17,22,24,25,26,27,28,35,41,45,48]	11

**Table 4:** Summary of Data Processing and Posture Recognition Approaches Identified in the Literature.

Algorithmic Approach	References from Included Studies	Total Studies
**Heuristic & Threshold-based Methods** (Simple rules, kinematics)	[13,15,22,23,24,25,26,28,29,32,33,37,39,40,41,42,48]	17
**Traditional Machine Learning** (SVM, KNN, Random Forest)	[16,17,18,30,31,35,47]	7
**Deep Learning Models** (CNN, LSTM, CNN-LSTM)	[14,27,34,36]	4

**Table 5:** Summary of User Feedback and Interaction strategies identified in the literature.

Feedback Modality	References from Included Studies	Total Studies
**Vibrotactile Feedback** (Real-time physical alerts)	[16,17,22,23,24,36,45,48]	8
**Visual Feedback** (Mobile apps, Dashboards)	[16,22,24,26,28,32,33,38,41,45]	10
**Auditory Feedback** (Hands-free notifications)	[25,36,45]	3
**Offline Analytical Feedback** (Post-hoc clinical analysis)	[13,14,15,18,27,29,30,31,34,37,38,39,40,42,47]	15

**Table 6:** Summary of Energy Management and Harvesting strategies identified in the literature.

Energy Strategy	References from Included Studies	Total Studies
**Low-Power Firmware & Communication** (Adaptive sampling, Edge computing, Duty-cycling)	[17,32,45,47]	4
**Piezoelectric & Triboelectric Harvesting** (Friction/vibration to power generation)	[12]	1
**Inductive & Electromagnetic Generation** (Resonant coils and inductive patches)	[13]	1

## Data Availability

No new datasets were generated or analyzed during the current study. The study is based on a systematic review of the published literature.

## Abbreviations

    The following abbreviations are used in this manuscript:

ANN	Artificial Neural Network
BLE	Bluetooth Low Energy
CINAHL	Cumulative Index to Nursing and Allied Health Literature
CNN-LSTM	Convolutional Neural Network – Long Short-Term Memory
ECG	Electrocardiogram
FBG	Fiber Bragg Grating
ID	Identification
IMU/IMUs	Inertial Measurement Unit(s)
IoT	Internet of Things
KNN	K-Nearest Neighbors
LSTM	Long Short-Term Memory
MoCap	Motion Capture
MSD/MSDs	Musculoskeletal Disorder(s)
PCA	Principal Component Analysis
PCB	Printed Circuit Board
PCC	Population, Concept, Context
PPE	Personal Protective Equipment
PPy	Polypyrrole
PRISMA	Preferred Reporting Items for Systematic Reviews and Meta-Analyses
PVDF	Polyvinylidene Fluoride
PZT	Lead Zirconate Titanate
SVM	Support Vector Machine
WMSD/WMSDs	Work-related Musculoskeletal Disorder(s)
WRMSD/WRMSDs	Work-related Musculoskeletal Disorder(s)

## Appendix A. Database Search Strategies

### Appendix A.1. Scopus

TITLE-ABS-KEY((people OR person OR user OR professional* OR “worker*” OR

“occupational*” OR “industrial*” OR “manufactur*” OR “logistic*” OR

“nurs*” OR “healthcare staff” OR “emergency servic*” OR “manual labor”

OR “operator*” OR “staff member*” OR “employee*” OR “laborer*”) AND

(“smart textile*” OR “e-textile*” OR “smart garment*” OR “smart cloth*”

OR “sensor* textile” OR “sensor-embedded fabric*” OR

“textile-based sensor*” OR “wearable textile*” OR

“fabric-integrated sensor*” OR “conductive fabric*” OR

“wearable garment*” OR “body-worn textile*” OR “body worn sensor*” OR

“smart fabric*” OR “e-fabric*” OR “sensor* fabric” OR

“textile sensor*” OR “sensor-integrated textile*” OR

“conductive textile*” OR “electronic textile*” OR

“sensor-embedded clothing” OR “sensor-embedded textile*”) AND

(“musculoskeletal disorder*” OR “musculoskeletal discomfort” OR

“work-related musculoskeletal” OR “ergonomic risk*” OR

“ergonomic assessment” OR “occupational health” OR “postural health” OR

“workplace safety” OR “posture monitoring” OR “posture detection” OR

posture OR “musculoskeletal injur*” OR “musculoskeletal injury*” OR

“MSD” OR “MSDs” OR “work-related musculoskeletal disorder*” OR “WMSD*”

OR “occupational musculoskeletal disorder*” OR

“repetitive strain injur*” OR “workplace musculoskeletal disorder*” OR

“Cumulative Trauma Disorder*” OR “spinal disorder*” OR

“occupational-related injury*” OR “back injury*” OR “ergonomic*” OR

“back pain” OR “occupational disease*” OR injur* OR

“musculoskeletal pain” OR “musculoskeletal syndrome” OR

“postural discomfort” OR “spinal injury*”))

### Appendix A.2. Web of Science

TS=(

 (people OR person OR user OR professional* OR worker* OR occupational*

 OR industrial* OR manufactur* OR logistic* OR nurs* OR “healthcare staff”

 OR “emergency servic*” OR “manual labor” OR operator* OR “staff member*”

 OR employee* OR laborer*)

 AND

 (“smart textile*” OR “e-textile*” OR “smart garment*” OR “smart cloth*”

 OR “sensor* textile” OR “sensor-embedded fabric*”

 OR “textile-based sensor*” OR “wearable textile*”

 OR “fabric-integrated sensor*” OR “conductive fabric*”

 OR “wearable garment*” OR “body-worn textile*” OR “body worn sensor*”

 OR “smart fabric*” OR “e-fabric*” OR “sensor* fabric”

 OR “textile sensor*” OR “sensor-integrated textile*”

 OR “conductive textile*” OR “electronic textile*”

 OR “sensor-embedded clothing” OR “sensor-embedded textile*”)

 AND

 (“musculoskeletal disorder*” OR “musculoskeletal discomfort”

 OR “work-related musculoskeletal” OR “ergonomic risk*”

 OR “ergonomic assessment” OR “occupational health”

 OR “postural health” OR “workplace safety”

 OR “posture monitoring” OR “posture detection” OR posture

 OR “musculoskeletal injur*” OR “musculoskeletal injury*”

 OR MSD OR MSDs OR “work-related musculoskeletal disorder*”

 OR WMSD* OR “occupational musculoskeletal disorder*”

 OR “repetitive strain injur*” OR “workplace musculoskeletal disorder*”

 OR “Cumulative Trauma Disorder*” OR “spinal disorder*”

 OR “occupational-related injury*” OR “back injury*”

 OR ergonomic* OR “back pain” OR “occupational disease*”

 OR injur* OR “musculoskeletal pain” OR “musculoskeletal syndrome”

 OR “postural discomfort” OR “spinal injury*”)

)

### Appendix A.3. PubMed

(

 people[tiab] OR person[tiab] OR user[tiab] OR professional*[tiab]

 OR worker*[tiab] OR occupational*[tiab] OR industrial*[tiab]

 OR manufactur*[tiab] OR logistic*[tiab] OR nurs*[tiab]

 OR “healthcare staff”[tiab] OR “emergency servic*”[tiab]

 OR “manual labor”[tiab] OR operator*[tiab]

 OR “staff member*”[tiab] OR employee*[tiab] OR laborer*[tiab]

)

AND

(

 “smart textile*”[tiab] OR “e-textile*”[tiab]

 OR “smart garment*”[tiab] OR “smart cloth*”[tiab]

 OR “sensor* textile”[tiab] OR “sensor-embedded fabric*”[tiab]

 OR “textile-based sensor*”[tiab] OR “wearable textile*”[tiab]

 OR “fabric-integrated sensor*”[tiab] OR “conductive fabric*”[tiab]

 OR “wearable garment*”[tiab] OR “body-worn textile*”[tiab]

 OR “body worn sensor*”[tiab] OR “smart fabric*”[tiab]

 OR “e-fabric*”[tiab] OR “sensor* fabric”[tiab]

 OR “textile sensor*”[tiab] OR “sensor-integrated textile*”[tiab]

 OR “conductive textile*”[tiab] OR “electronic textile*”[tiab]

 OR “sensor-embedded clothing”[tiab]

 OR “sensor-embedded textile*”[tiab]

)

AND

(

 “musculoskeletal disorder*”[tiab]

 OR “musculoskeletal discomfort”[tiab]

 OR “work-related musculoskeletal”[tiab]

 OR “ergonomic risk*”[tiab] OR “ergonomic assessment”[tiab]

 OR “occupational health”[tiab] OR “postural health”[tiab]

 OR “workplace safety”[tiab] OR “posture monitoring”[tiab]

 OR “posture detection”[tiab] OR posture[tiab]

 OR “musculoskeletal injur*”[tiab]

 OR “musculoskeletal injury*”[tiab] OR MSD[tiab] OR MSDs[tiab]

 OR “work-related musculoskeletal disorder*”[tiab]

 OR WMSD*[tiab] OR “occupational musculoskeletal disorder*”[tiab]

 OR “repetitive strain injur*”[tiab]

 OR “workplace musculoskeletal disorder*”[tiab]

 OR “Cumulative Trauma Disorder*”[tiab]

 OR “spinal disorder*”[tiab] OR “occupational-related injury*”[tiab]

 OR “back injury*”[tiab] OR ergonomic*[tiab]

 OR “back pain”[tiab] OR “occupational disease*”[tiab]

 OR injur*[tiab] OR “musculoskeletal pain”[tiab]

 OR “musculoskeletal syndrome”[tiab]

 OR “postural discomfort”[tiab] OR “spinal injury*”[tiab]

)

### Appendix A.4. CINAHL

(

 TI (people OR person OR user OR professional* OR worker* OR occupational*

 OR industrial* OR manufactur* OR logistic* OR nurs* OR “healthcare staff”

 OR “emergency servic*” OR “manual labor” OR operator* OR “staff member*”

 OR employee* OR laborer*)

 OR

 AB (people OR person OR user OR professional* OR worker* OR occupational*

 OR industrial* OR manufactur* OR logistic* OR nurs* OR “healthcare staff”

 OR “emergency servic*” OR “manual labor” OR operator* OR “staff member*”

 OR employee* OR laborer*)

)

AND

 TI (“smart textile*” OR “e-textile*” OR “smart garment*” OR “smart cloth*”

 OR “sensor* textile” OR “sensor-embedded fabric*”

 OR “textile-based sensor*” OR “wearable textile*”

 OR “fabric-integrated sensor*” OR “conductive fabric*”

 OR “wearable garment*” OR “body-worn textile*” OR “body worn sensor*”

 OR “smart fabric*” OR “e-fabric*” OR “sensor* fabric”

 OR “textile sensor*” OR “sensor-integrated textile*”

 OR “conductive textile*” OR “electronic textile*”

 OR “sensor-embedded clothing” OR “sensor-embedded textile*”)

 OR

 AB (“smart textile*” OR “e-textile*” OR “smart garment*” OR “smart cloth*”

 OR “sensor* textile” OR “sensor-embedded fabric*”

 OR “textile-based sensor*” OR “wearable textile*”

 OR “fabric-integrated sensor*” OR “conductive fabric*”

 OR “wearable garment*” OR “body-worn textile*” OR “body worn sensor*”

 OR “smart fabric*” OR “e-fabric*” OR “sensor* fabric”

 OR “textile sensor*” OR “sensor-integrated textile*”

 OR “conductive textile*” OR “electronic textile*”

 OR “sensor-embedded clothing” OR “sensor-embedded textile*”)

)

AND

(

 TI (“musculoskeletal disorder*” OR “musculoskeletal discomfort”

 OR “work-related musculoskeletal” OR “ergonomic risk*”

 OR “ergonomic assessment” OR “occupational health”

 OR “postural health” OR “workplace safety” OR “posture monitoring”

 OR “posture detection” OR posture OR “musculoskeletal injur*”

 OR “musculoskeletal injury*” OR MSD OR MSDs

 OR “work-related musculoskeletal disorder*” OR WMSD*

 OR “occupational musculoskeletal disorder*”

 OR “repetitive strain injur*” OR “workplace musculoskeletal disorder*”

 OR “Cumulative Trauma Disorder*” OR “spinal disorder*”

 OR “occupational-related injury*” OR “back injury*” OR ergonomic*

 OR “back pain” OR “occupational disease*” OR injur*

 OR “musculoskeletal pain” OR “musculoskeletal syndrome”

 OR “postural discomfort” OR “spinal injury*”)

 OR

 AB (“musculoskeletal disorder*” OR “musculoskeletal discomfort”

 OR “work-related musculoskeletal” OR “ergonomic risk*”

 OR “ergonomic assessment” OR “occupational health”

 OR “postural health” OR “workplace safety” OR “posture monitoring”

 OR “posture detection” OR posture OR “musculoskeletal injur*”

 OR “musculoskeletal injury*” OR MSD OR MSDs

 OR “work-related musculoskeletal disorder*” OR WMSD*

 OR “occupational musculoskeletal disorder*”

 OR “repetitive strain injur*” OR “workplace musculoskeletal disorder*”

 OR “Cumulative Trauma Disorder*” OR “spinal disorder*”

 OR “occupational-related injury*” OR “back injury*” OR ergonomic*

 OR “back pain” OR “occupational disease*” OR injur*

 OR “musculoskeletal pain” OR “musculoskeletal syndrome”

 OR “postural discomfort” OR “spinal injury*”)

)

## Appendix B. Summary of Included Studies

This section presents a description of the 34 articles selected for the literature review, including the author(s), year, country of publication, objectives, methodology, and results, as well as the conclusions and limitations of each study, as illustrated in Table A1. This comprehensive overview enables a deeper understanding of the solutions explored in recent years and their implementations, while also identifying potential shortcomings and opportunities for future improvements.

**Table A1:** Description of the 34 articles selected using the PRISMA-ScR method.

ID	Author(s), Year, Country	Objectives	Methodology and Results	Conclusions and Limitations	Relevance to Occupational Posture Monitoring
S1 [13]	Monti, G., Lezzi, D., Tarricone, L., 2025, Italy	To demonstrate the feasibility of a wearable system based on resonant inductive coupling for monitoring neck posture, detecting inclinations through variations in transmission parameters.	Proof-of-concept study based on a textile resonator prototype for neck-posture monitoring. Further real-world validation was identified as necessary.	The system is promising, lightweight, and low-power, but circuit miniaturization is needed for real-world use. Future work includes validation in real environments and improved integration into clothing.	Neck inclination is relevant to occupational ergonomic assessment, particularly in tasks involving sustained or repeated neck flexion. The study contributes a textile-integrated sensing approach, although its effectiveness in occupational settings requires further validation.
S2 [16]	Hou, Y., Wang, Z., Liu, H., Xia, M., Fan, X., Ye, Q., 2025, China	Create an intelligent garment that monitors and recognizes sitting postures, encouraging adjustments to prevent back problems.	Data from twenty participants using machine learning (best results with Random Forest) and usability testing showed over 95% posture recognition accuracy, improved posture, and high user acceptance.	The garment effectively promotes posture changes and prevents discomfort, with positive feedback, but faces challenges in integrating comfortable electronics, personalizing feedback, and hardware limitations.	The system directly addresses sitting-posture monitoring and corrective feedback, which are relevant to sedentary occupations and tasks involving prolonged sitting. Its practical effectiveness across different workplaces remains to be established.
S3 [17]	Selvaraj, V., Nagaraj, A., Whiffen, B.G., Min, S., 2024, United States	Create a wearable sensor system to monitor lifting risks and use machine learning to predict potential injuries.	The system uses IMU sensors on wrists and lower back at 400 Hz to classify lifting risks with an F1 score of 0.89, providing feedback to users.	The system is accurate and helpful for injury prevention in lifting, but faces limitations in sensor synchronization, communication bandwidth, and hub reliability.	The study directly addresses occupationally relevant lifting movements and injury risk. Wrist and lower-back motion measurements can support ergonomic assessment of manual material handling tasks.
S4 [32]	Aliyana, A.K., Yang, D., Tangsirinaruenart, O., Stylios, G.K., 2024, United Kingdom	Create a textile stretch sensor integrated into sportswear with Internet of Things (IoT) connectivity for real-time movement monitoring, helping improve performance and prevent injuries.	The textile sensors were tested in a controlled gym environment for real-time movement monitoring.	Textile sensors are effective and affordable for sports monitoring, enhancing training and injury prevention, but face limitations like higher hysteresis in larger sizes, limited range, and the need for ongoing calibration.	The study demonstrates textile-based strain sensing during body movements. Although evaluated in a sports context, the sensing approach may inform the development of wearable systems for monitoring occupational movements; direct occupational applicability was not assessed.
S5 [33]	Yang, C.-M., Wu, S.-C., Liao, K.-C., Wu, Z.-W., Chen, S.-H., Chen, C.-C., 2024, Taiwan	Create a smart garment that detects posture and breathing in real time to improve activities like yoga.	The system was tested under controlled conditions for real-time posture and breathing monitoring during yoga-related activities.	The garment offers real-time monitoring and feedback to aid posture correction and exercise improvement, but it requires validation in diverse environments as tests were conducted under controlled conditions.	The study evaluates smart-garment sensing for posture detection and breathing monitoring. Its relevance lies in the technological approach to posture recognition, although applicability to occupational tasks was not directly evaluated.
S6 [15]	Tian, B., Zheng, K., Yu, R., Chen, X., Guo, P., Wu, Y., Liang, J., Wu, W., 2023, China	Create and optimize high-performance, durable, and low-cost e-textiles with sensors for human movement and posture monitoring.	Developed composite inks and printed sensors, with the optimized version showing high sensitivity, durability, and effectiveness in posture recognition.	The ink formulation is crucial for performance, enabling reliable and comfortable e-textiles, though further real-world validation and scalable manufacturing are necessary.	The development of durable, printed textile sensors supports the technological basis for wearable movement and posture monitoring. Their suitability for occupational use would require evaluation under representative workplace conditions.
S7 [14]	Liang, Z., Zhang, D., Xu, G., Xie, F., Cai, H., Guo, H., Cheng, J., 2023, China	Create an intelligent garment that monitors pressure on nearly the entire body.	Using conductive wires and pressure-sensitive materials sewn into fabric with an efficient reading system, the sensors cover 80% of the body, are durable, and accurately recognize actions and postures.	The garment is suitable for long-term monitoring but requires validation in daily life and the development of specific algorithms.	The garment demonstrates extensive body-pressure sensing and posture recognition. These capabilities may support future ergonomic monitoring of body position and contact pressure, although occupational applications were not directly evaluated.
S8 [12]	Mokhtari, F., Cheng, Z., Wang, C.H., Foroughi, J., 2023, Australia	To develop piezoelectric sensors for real-time safety and health monitoring of industrial workers.	Using ceramics and polymers fabricated through 3D printing, electrospinning, and melt spinning, the sensors demonstrated high sensitivity, durability, and autonomous energy generation for monitoring tasks.	Piezoelectric energy harvesting is promising for wearables, but challenges in durability, environmental resistance, standardization, and scaling remain.	The study explicitly targets industrial-worker monitoring and contributes wearable sensing and energy-harvesting technologies that may support continuous occupational safety and ergonomic monitoring.
S9 [34]	Lin, Q., Peng, S., Wu, Y., Liu, J., Jia, H., Hu, W., Hassan, M., Seneviratne, A., Wang, C.H., 2023, Sweden	Create a loose-fitting smart garment for high-accuracy human activity recognition.	Sensitive sensors integrated into clothing, combined with CNN-LSTM models and domain adaptation, achieved over 91% accuracy for known users and 82.9% for new users, with sensor placement affecting performance.	The system effectively detects activities and postures, useful for health monitoring, but faces challenges in data diversity, energy efficiency, washability, and daily robustness.	The study contributes to wearable activity and posture recognition using loose-fitting garments. Its relevance is primarily technological, as transferability to occupational movement monitoring was not directly evaluated.
S10 [26]	Ahmmed, A.S., Teyeme, Y., Malengier, B., Tesfaye, T., Daba, H., Esmelealem, K., Van Langenhove, L.V., 2022, Belgium	Create a smart shirt that monitors trunk posture in real time during activities like cycling.	The methodology developed textile lines with magnetic sensors, calibrated and wash-resistant, capable of accurately measuring trunk angles, with an average of 21.5°.	The shirt monitors posture during movement and is useful for athletes and health professionals, though improvements are needed for sensor attachment and durability.	The system measures trunk orientation and bending angles using a smart shirt. These biomechanical variables are relevant to occupational ergonomic assessment, although the system was evaluated during cycling rather than occupational tasks.
S11 [29]	Pinnelli, M., Pulcinelli, M., Carnevale, A., Di Tocco, J., Massaroni, C., Schena, E., Longo, U.G., Denaro, V., 2022, Italy	To create and test a textile wearable system capable of detecting lumbar hyperlordosis in dancers, aiming to prevent back pain.	The study used MoCap and textile sensors tested on dancers to detect hyperlordosis, successfully distinguishing hyperlordotic from correct postures and demonstrating effective lumbar posture monitoring.	The textile sensor system effectively monitors lumbar hyperlordosis and may help prevent back pain, but further research is needed to adapt it for more users and movements.	The study investigates textile-based detection of lumbar hyperlordosis and compensatory lumbar posture. These measurements may inform the assessment of spinal posture in occupational settings, although the study focused on ballet dancers and did not directly evaluate work-related ergonomic risks.
S12 [37]	Sikligar, D., Nguessan, L., Pham, D., Grupper, J., Beaudette, A., Ling, A., Walsh, C., Golecki, H.M., 2022, United States	To create a shirt with embedded textile sensors for continuous posture monitoring, helping to detect poor posture and prevent pain.	Resistive and capacitive sensors in T-shirts were tested for posture detection; resistive sensors proved more sensitive and stable, effectively distinguishing between good and bad postures, and remained functional after washing.	Textile sensors are promising for continuous posture monitoring and research, but face challenges like user variability, calibration, and limited testing.	The system directly addresses continuous posture monitoring and the identification of poor postures. Its sensing and garment-integration approaches are relevant to the development of occupational ergonomic monitoring systems.
S13 [30]	Chen, Z., Chen, X., Ma, Y., Guo, S., Qin, Y., Liao, M., 2021, China	To identify the best sensors for recognizing different arm movements, highlighting those with the highest discrimination ability.	Data from one participant was analyzed using distance measures and PCA. Euclidean distance effectively distinguished movements, especially on the arm.	Sensors on the arm with Euclidean distance work well for motion recognition, but the study was limited by a single participant and few movements. Future work should include more patterns and advanced methods.	Arm-movement recognition is relevant to tasks involving repetitive or sustained upper-limb movements. The study informs sensor placement and movement-discrimination methods, although its small sample limits generalizability.
S14 [38]	Kim, H., Shaqeel, A., Han, S., Kang, J., Yun, J., Lee, M., Lee, S., Kim, J., Noh, S., Choi, M., Lee, J., 2021, South Korea	To create sensitive, flexible, and safe fiber sensors using in situ formed silver nanoparticles for smart clothing applications.	The sensors, created by silver absorption and reduction in polyurethane fibers, offer high sensitivity, wide strain range, and durability, making them ideal for movement monitoring.	The safe-material fabrication produced effective sensors promising for smart textiles and motion tracking, though further real-world testing and long-term validation are needed.	The study contributes flexible fiber-sensor technology for monitoring body movement. Such sensing capabilities may support occupational wearable systems, although occupational postures and tasks were not directly assessed.
S15 [41]	Alfaqawi, M., Ben Mabrouk, M.B., Davai, S., 2021, France	To develop a hybrid system that combines observation and instrument-based methods to assess operators’ postures in real time, aiding in the prevention of work-related musculoskeletal disorders.	A smart garment with sensors and cameras was designed to classify postures into risk levels. During testing with an operator, the system correlated data in real time, providing accurate visual analysis.	The hybrid system assesses posture risks in real time, but further validation with more operators and environments is needed.	The study directly targets operators’ postures and work-related musculoskeletal risk. Combining wearable sensing with visual observation may support workplace posture assessment and ergonomic risk classification.
S16 [27]	Petz, P., Eibensteiner, F., Langer, J., 2021, Austria	Create a smart shirt that monitors body posture and movements to improve ergonomics and safety.	Using a shirt with inertial sensors and machine learning, the system identifies movements and postures with over 80% accuracy, aiding injury prevention.	The shirt is feasible, flexible, and useful for real-time ergonomic monitoring, despite challenges with electrical resistance, energy consumption, and validation in real environments.	The system directly addresses ergonomic monitoring and injury prevention through wearable posture and movement recognition. Further validation in real workplaces is needed to establish its generalizability.
S17 [42]	Zaltieri, M., Lo Presti, D.L., Massaroni, C., Schena, E., D’Abbraccio, J., Massari, L., Oddo, C.M., Formica, D., Caponero, M.A., Bravi, M., Miccinilli, S., Sterzi, S., 2020, Italy	To evaluate whether a wearable system with FBG sensors can monitor dorsal flexion and extension movements to prevent back pain in video terminal workers.	A T-shirt with silicone patches containing FBG sensors was developed and tested with six volunteers performing flexion and extension movements, demonstrating high sensitivity and accuracy, aligning well with a reference system.	The system is effective for monitoring spinal movements and preventing postural issues, but requires further validation with more participants.	The study directly addresses spinal movement monitoring among video-terminal workers. Dorsal flexion and extension measurements are relevant to assessing postural exposure in sedentary work.
S18 [40]	Patiño, A., Khoshnam, M., Menon, C., 2020, Switzerland	Create a wearable device to monitor trunk movements and prevent back pain.	A T-shaped sensor sewn into elastic clothing was calibrated and tested with a participant performing various movements. It accurately detected forward bending with stable readings and minimal interference.	The system is comfortable, lightweight, and effective for monitoring spinal movements to aid in pain prevention. However, the study was conducted with only one participant, and further testing with more people is needed.	The system measures trunk movements, particularly forward bending, which is relevant to occupational ergonomic assessment. However, the reported validation involved only one participant and requires further testing.
S19 [22]	Bootsman, R., Markopoulos, P., Qi, Q., Wang, Q., Timmermans, A.A., 2019, Netherlands	Create a smart garment to help nurses avoid postures that cause back pain.	Through iterative development and user testing, the system accurately detects lumbar flexion, is comfortable, and helps reduce poor postures in the short term.	The garment is effective for posture monitoring, but more research is needed for long-term use due to limitations in sample size, duration, durability, and comfort.	The study directly addresses occupational posture monitoring in nursing. Detection of lumbar flexion and feedback to users are relevant to reducing potentially adverse postures during patient-care activities.
S20 [43]	Rohit, A., Kelestemur, Y., Runyon, J.C., Kaya, S., 2019, United States	Create wearable patches with multiple sensors to monitor posture, movements, and health, using data fusion for accurate analysis.	The patches, developed with nanocomposite materials and integrated sensors, are sensitive and durable, effectively distinguishing physical activities and biomedical signals, enhancing event detection accuracy.	The patches are compact, affordable, and effective for continuous health and performance monitoring, but require further improvements in data fusion and validation in larger populations for greater robustness.	The study contributes wearable multisensor and data-fusion approaches for monitoring movement and posture. Their potential use in occupational settings is indirect because workplace-specific ergonomic assessment was not directly evaluated.
S21 [44]	Rohit, A., Kelestemur, Y., Runyon, J.C., Kaya, S., 2019, United States	Develop wearable patches with multiple sensors to monitor posture, movements, and health, using data fusion.	Using elastomeric materials and conductive textiles, the patches integrate sensors and data-fusion algorithms, making them sensitive, durable, and capable of identifying activities and biomedical signals.	They are compact, affordable, and efficient for continuous monitoring with various applications, but need improvements in data-fusion algorithms and validation with larger populations.	The study contributes to the development of textile-integrated multisensor patches for movement monitoring. The sensing and data-fusion methods may inform occupational applications, although workplace-specific validation was not reported.
S22 [23]	Barone, V.J., Yuen, M.C., Kramer-Boniglio, R., Sienko, K.H., 2019, United States	Create a wearable system with flexible sensors and vibrotactile feedback to monitor and improve seated lumbar posture in real time.	The sensor was validated with five participants, showing improvements in posture and comfortable vibratory feedback.	The system shows potential for lumbar posture training and monitoring but is limited by a small sample size and testing conditions.	The system directly addresses seated lumbar posture and feedback, which are relevant to occupations involving prolonged sitting. Its effectiveness requires validation in larger and more representative workplace populations.
S23 [45]	Vega-Barbas, M., Diaz-Olivares, J.A., Lu, K., Forsman, M., Seoane, F., Abtahi, F., 2019, Sweden	Develop a platform to monitor ergonomic risks using wearable sensors and edge computing.	The architecture with sensors, app, and servers was tested in real scenarios, collecting millions of measurements. The system demonstrates acceptable usability and supports up to 500 users.	The system shows potential for continuous, personalized assessments, though improvements are needed. Limitations such as comfort, integration, and user acceptance are still pending, with long-term studies to be conducted.	The platform explicitly targets ergonomic-risk monitoring using wearable sensors and computing infrastructure. It may support continuous assessment at scale, although further evidence is needed on comfort, integration, and long-term workplace use.
S24 [18]	Skach, S., Stewart, R., Healey, P.G.T., 2018, United Kingdom	Developing pants with sensors to automatically detect sitting postures.	Sensor-equipped pants and machine learning were used to collect data from six people across 19 postures, achieving over 99% accuracy in classifying postures.	The system has potential to discreetly monitor behavior and attention, but limitations include data collected in a controlled environment with few participants, requiring real-world testing.	The study demonstrates textile-integrated recognition of sitting postures. The technology may be transferable to occupational settings involving prolonged sitting, although workplace-specific ergonomic outcomes were not evaluated.
S25 [48]	Caya, M.V.C., Sejera, M.P., Deriquito, J.P.P., Losaria, N.F.F., Manzano, M.J.C., 2018, Philippines	To develop a wearable device using e-textile technology to detect and monitor poor postures in golf players, beyond just slouching positions.	A prototype with accelerometers, a flex sensor, and a microcontroller was developed and tested in a controlled environment, successfully monitoring posture and swings.	The system effectively detects poor postures in golfers, but it does not offer real-time monitoring. It was tested with few participants in a controlled setting, and power consumption was not analyzed.	The study uses e-textile technology and wearable sensors to monitor posture during golf swings. Its contribution concerns the technical feasibility of wearable posture monitoring; its relevance to occupational ergonomic assessment is indirect, as occupational tasks were not evaluated.
S26 [24]	Du, J., Wang, Q., de Baets, L., Markopoulos, P., 2017, Netherlands	Create and test a wearable system that monitors shoulder posture and encourages corrections and exercises to prevent pain.	The system effectively reduced poor postures, had good usability, and was well received, with some suggestions for improvements.	The system shows potential for improving posture and supporting musculoskeletal pain prevention in sedentary work, but it requires long-term studies and improvements in aesthetics and feedback.	The study addresses shoulder posture and corrective feedback in sedentary work. Its findings are directly relevant to occupational interventions aimed at reducing adverse shoulder postures and associated musculoskeletal discomfort.
S27 [35]	Durbhaka, G.K., 2016, India	To develop an intelligent fabric that monitors posture and temperature, automatically adjusting to provide comfort during sleep and in various environmental conditions.	The methodology involved creating a prototype with sensors and actuators to classify postures and adjust the fabric, achieving an initial accuracy of 87%, demonstrating its ability to improve comfort automatically.	The fabric can adapt to various postures and conditions, providing comfort during sleep, but improvements are needed in accuracy and prototype development due to limited data.	The study integrates posture sensors and actuators into adaptive smart fabric to detect body posture during sleep. Although its application is non-occupational, it demonstrates textile-integrated posture sensing and adaptive responses that may inform the development of wearable systems for other contexts.
S28 [39]	Ahmadizadeh, C., Galli, V., Luder, V., Menon, C., 2024, Switzerland	Create textile inductive sensors to monitor back movements in a smart garment.	Sensors were designed and tested for sensitivity and stability, then integrated into a garment. They effectively detected back movements with good sensitivity and low drift.	Inductive textile sensors are promising for comfortable movement monitoring with health applications, but further validation with more participants and complex movements is needed due to limited testing.	The system monitors back movements using textile-integrated inductive sensors. Its sensing capabilities may support occupational spinal-movement assessment, although the study’s application to workplace tasks was not directly evaluated.
S29 [31]	Tan, Y.Y., Gupta, U., Chia, P.Z., Pambudi, C.P., Lim, B.E.H., Soh, G.S., Low, H.Y., 2025, Singapore	Create a smart knitted shirt that monitors arm movements and predicts muscle fatigue.	The sensors effectively tracked movements and fatigue, with the model producing accurate estimates closely matching Vicon data.	The shirt monitors movement and fatigue non-invasively, but needs more testing and validation.	Arm-motion and muscle-fatigue monitoring may be relevant to repetitive or physically demanding occupational tasks. The study provides a technological basis for such applications, although workplace-specific performance was not directly assessed.
S30 [36]	Wang, Q., Cui, F., Zhang, R., Chen, L., Yuan, J., Sun, X., Yu, B., 2024, Netherlands	Develop a smart legging with textile strain sensors and an LSTM model to monitor lower-limb motion, classify running postures, and offer feedback to prevent knee injuries.	This study developed a textile-based sensor system that accurately classifies running postures with 99.1% accuracy and supports posture correction, while being comfortable and user-friendly.	The study highlights the potential of fabric strain sensors for real-time posture monitoring with smart leggings, though it notes some material and testing limitations.	The study uses textile strain sensors to monitor lower-limb movements and classify running postures. Its contribution concerns textile-based movement classification, although applicability to occupational tasks was not directly evaluated.
S31 [28]	Eklund, J., Forsman, M., 2019, Sweden	Smart work clothes to automate musculoskeletal disorder (MSD) risk assessment via postures, movements, heart rate, and breathing measurements.	A textile vest with sensors and IMUs was used for ECG, breathing, posture, movement, and heart-rate monitoring; it was comfortable, suitable for full-day wear, and accepted by users.	A promising low-cost tool for risk assessment, training, and workplace improvements. It requires validity studies, incorporation of hand/foot sensors, and ethical data handling.	The study directly targets occupational MSD risk assessment through wearable monitoring of posture, movement, and physiological parameters. It also addresses practical considerations for full-day workplace use.
S32 [25]	Mahdavian, N., Lind, C.M., Olivares, J.A.D.I.A.Z., Pascual, A.I.R.I.O.N.D.O., Högberg, D., Brolin, E., Yang, L., Forsman, M., Hanson, L., 2018, Sweden	Test smart-textile feedback to reduce adverse arm postures (≥60°) in assembly and prevent work-related musculoskeletal disorders.	Twenty-four trainees performed assembly tasks in a laboratory-simulated occupational setting using IMU T-shirts. The virtual coach significantly reduced arm movement time by 43 seconds compared with baseline, outperforming no feedback and human coaching.	Automatic feedback improves work techniques for efficiency and health, but the findings are based on a small sample and require further investigation of persistence and workstation effects.	The study directly evaluates textile-based feedback for adverse arm postures during assembly tasks. Its findings are relevant to occupational ergonomic interventions, although further studies are needed to assess long-term effects and performance across workstations.
S33 [46]	Esfahani, M.I.M., Taghinedjad, S., Mottaghitalab, V., Narimani, R., Parnianpour, M., 2016, Iran	Develop a printed polypyrrole (PPy)-coated nylon/lycra strain sensor for body posture and movement measurement in biomechanics, including joints, sports, and rehabilitation.	Screen printing with chemical vapour deposition of polypyrrole on nylon/lycra showed good sensitivity, linearity, and stability with minimal rate dependence.	The study developed a wearable motion sensor that is easier to print and suitable for large areas, but it still needs testing on actual joints, more cycles, and formulation improvements.	The study develops and calibrates a printed textile strain sensor for measuring human movement orientation. Its contribution lies in wearable sensing technology for biomechanical movement measurement, which may support future occupational posture-monitoring applications.
S34 [47]	Mokhlespour Esfahani, M.I., Nussbaum, M.A., Kong, Z. (James), 2019, USA	Classify nine manual material-handling tasks using smart textiles to assess occupational physical exposures and prevent work-related musculoskeletal disorders.	Eleven participants performed manual material-handling tasks using smart socks and undershirts with strain gauges. The classification methods achieved up to 97.7% accuracy overall, with the best results from the undershirt.	The smart textile system is viable for manual material-handling classification in workplaces, aiding ergonomic interventions. However, the findings are based on laboratory simulations with limited tasks and participants and lack long-term field validation.	The study directly evaluates smart textiles for classifying occupational manual material-handling tasks. It supports the use of wearable sensing to assess physical exposure and inform ergonomic interventions.
